# Health of the Elderly Migration Population in China: Benefit from Individual and Local Socioeconomic Status?

**DOI:** 10.3390/ijerph14040370

**Published:** 2017-04-01

**Authors:** Qing Wang

**Affiliations:** School of Business, Dalian University of Technology, No. 2 Dagong Road, Panjin 124221, China; qingwang@dlut.edu.cn or wangqing1984@126.com; Tel.: +86-0427-263-1979

**Keywords:** elderly migrant, health, income, local socioeconomic status, public service provision, China

## Abstract

The study aims to estimate the relationship between the individual/local socioeconomic status and the health of internal elderly migrants in China. A multilevel logistic model was used to estimate this association. The estimations were undertaken for 11,111 migrants aged over 60 years, using nationally representative data: the 2015 Migrant Dynamics Monitoring Survey (MDMS), which was carried out in China. Odds ratios with 95% confidence intervals were reported. Both the household income per capita and the area-level average wage were positively associated with migrants’ self-reported health; however, public service supply was not significantly related to their health. In addition, given the household income, migrants living in communities with a higher average wage were more likely to report poor health. Migrants’ health benefited from individual socioeconomic status, but not from the local socioeconomic status, which the migrants cannot enjoy. This study highlights the importance of multilevel and non-discriminatory policies between migrants and local residents.

## 1. Introduction

Internal migration refers to the movement of people within a geopolitical unity, which is a common occurrence in many regions such as Europe, the US, and China [1]. China has the world’s largest migration population, which increased significantly from 50 million in 1990 to 252 million in 2014. During the last two decades, an increasing number of families migrated with their elderly members. The proportion of people aged over 45 years increased from 9.7% in 2010 to 12.9% in 2014, accounting for the overall increase in the number of migrants during this period [2].

The elderly migrants usually possess poorer socioeconomic characteristics. Over 30% of the elderly migrants, aged over 60 years, continue to work due to financial pressure. They usually accept low-paying jobs in labor-intensive industries, such as manufacturing, goods transportation, construction, entertainment, housekeeping, and restaurant services. In addition, without local *hukou* (local residents’ registration), migrants are not entitled to enjoy the same social benefits as the local people are. They are also less likely to have complete access to healthcare services, comfortable housing, and fair employment conditions [2,3,4]. 

The low socioeconomic status, combined with limited social benefits, has resulted in the unique characteristics in the measures for seeking medical treatment for the elderly migration population in China. According to the 2015 Migrant Dynamics Monitoring Survey (MDMS), 54.27% of the elderly migrants preferred being either self-treated or untreated rather than visiting hospitals; 18% requiring hospitalization did not use the inpatient service, and among those who received it, 30% returned to their hometown for hospitalization.

A robust socioeconomic gradient in health is well documented among the public, with higher socioeconomic statuses indicating better health outcomes [5,6]. However, studies on internal migrants provide inconsistent and scarce results. Studies using data from some high-income countries (e.g., Austria and the European Union) strengthened the evidence linking a lower socioeconomic status with poor health outcomes, whereas evidence from studies conducted in the US and Peru showed that individuals having low socioeconomic status had similar or better health than that of their richer and more acculturated counterparts [7,8,9,10,11,12,13,14,15]. 

To the best of my knowledge, only two studies about migrant workers’ health and its determinants have been conducted in China. Studies suggest that financial and employment difficulties contribute substantially to the mental health issues of migrant workers [16,17]. There is scarce information on Chinese migrants, particularly the elderly. Therefore, the association between health and the individual socioeconomic status for Chinese elderly migrants was evaluated accordingly.

Results regarding the health disparity in the socioeconomic status vary, possibly due to the differences in the study methodology and datasets. Further, the most important reason for this variation is that the mechanisms underlying the relationship between socioeconomic status and health may differ depending on the geographic location and sociocultural context of the population studied (e.g., social welfare system development) [10,14]. Thus, this study aims to estimate the extent to which the health of elderly migrants benefits from local socioeconomic status. The results showed that individual socioeconomic status was positively associated with migrants’ self-reported health, even controlling for the local socioeconomic status. Moreover, migrants’ health benefits only from the local socioeconomic status, which they are able to enjoy.

## 2. Methods

### 2.1. Data Source

A group of city-level variables and individual sample data were used to estimate the relationship between individual/local socioeconomic status and health of elderly migrants. Each city’s socioeconomic indicators were obtained from the 2016 China City Statistical Yearbook. Individual sample data for this study were drawn from the 2015 MDMS in China, carried out in the migrants’ destination cities. The survey was conducted in May 2015, commissioned by the National Population and Family Planning Commission of China, and coordinated by the China Population and Development Research Center. MDMS is an open access and nationally representative sample survey, and aims to examine the socioeconomic status of migrants, determinations of medical services use, and health outcomes.

The 2015 MDMS adopted a stratified three-stage probability proportionate to size (PPS) sampling, and the annual national data on migrants from each province in 2014 was considered the basic sampling frame. The survey covered 348 cities from all 32 provincial units in China. Within each city, communities were randomly selected using the PPS, resulting in 10,300 communities nationwide. In each selected community, 20 eligible individual migrants were randomly selected to participate in the survey. Migrants who moved across county boundaries and have been residing at their current location for more than one month were interviewed face-to-face by trained interviewers, using a structured questionnaire. All participants provided written consents. The MDMS sample is considered as a good representation of the population, with sampling errors being relatively small and within the acceptable range [18].

In total, 12,718 migrants aged over 60 years were interviewed. Of the total responses, 126 observations with either missing values or logical contradiction were excluded and 1481 observations without matching city-level data were deleted, after which the final sample size comprised 11,111 observations.

### 2.2. Variables

#### 2.2.1. Health Outcomes 

To measure the elderly migrants’ health, the MDMS includes the question: ‘How would you evaluate your health—healthy, basic healthy, or unhealthy?’ We constructed a category variable of ‘self-reported health’, the value of which ranges from 1 to 3 (1 = healthy; 2 = basic healthy; 3 = unhealthy). Self-reported health is a commonly used variable, and has been validated as a good indicator of overall health [19].

#### 2.2.2. Individual Socioeconomic Status

Individual socioeconomic status was measured by the logarithm (log) of household income per capita. Household income includes the wage income and operating income. The resources available to any family member depend on both the family’s income and the number of family members sharing that income. Therefore, the income was adjusted for family size by dividing by the number of family members. Family size refers to a census subfamily, which includes all related individuals in a household. 

#### 2.2.3. Local Socioeconomic Status

The health status of the migration population is influenced by both economic development, which was evaluated by average area-level wages, and the nature of public policies pursued by a government—that is, the quality and quantity of public services available to the population—which was measured by the log of public expenditure per capita [20].

#### 2.2.4. Individual and Migrate Characteristics

Demographic variables included gender (reference group: female), marital status (reference group: married with spouse present (common-law marriage was considered as being married)), the log of average daily exercise time, age, and age quadric. Insurance status was divided into uninsured, underinsured (reference group), and fully insured. If respondents reported that they had no insurance coverage, they were considered uninsured. The term ‘underinsured’ is defined as the status when migrants have medical insurance from places other than their place of residence. Medical expenses cannot be reimbursed by the local medical insurance institutions for underinsured migrants. Therefore, they do not have sufficient financial protection. Individuals with local insurance were regarded as the fully insured. Educational attainment in the data was defined at three levels: primary school or below, high school, and college or above, with primary school or below serving as the reference group.

Migrate characteristics include migrants’ reasons and scope for movements. Accordingly, the MDMS includes the question, ‘Why did you choose to move here?’ with the possible responses including the following: seeking job, looking after children or grandchildren, moving for retirement, keeping fit, or other reasons, which is a residual category asked in the survey. The variable for the move reasons was coded two if individuals moved to look after children or grandchildren, one if they move for retirement or for keeping fit, and zero if they moved to seek jobs or for other reasons. The move scope comprised two categories: intra-urban mobility and cross-city (reference group). 

### 2.3. Analytical Procedure

The descriptive statistics contain frequencies with percentages and means with standard deviations for the total sample, and by move reasons. Next, an ordered logit regression analysis controlling for income was conducted to estimate the correlation between income and health. Regression models were adjusted for age, gender, marital status, Han nationality, insurance status, education attainment, health behaviors, and region. Odds ratios (ORs) with 95% confidence intervals (CIs) were reported. Then, a two-level hierarchical ordered logit regression was formulated to estimate the association between individual/local socioeconomic status and health of the elderly migrants [21]. The local socioeconomic status and the interaction of individual and local socioeconomic status were controlled individually. 

An underlying endogeneity problem may exist if migrants move to a particular locality in order to achieve optimal health. Quasi-experimental or instrument variables may correct the bias [22]. However, because of data limitation, these corrections are unavailable. As alternative solutions, analyses were stratified by move reasons. Those migrants moving to look after children reach a particular locality wherein the family members reside with little consideration of their own optimal health. The analysis focusing on respondents who move to look after children may remove the endogeneity issue. Statistical analyses were performed using STATA 14.0 (StataCorp, College Station, TX, USA). 

## 3. Results

The descriptive statistics contain frequencies, as described in Table 1. The mean age of the participants was 67 years, 52% were male, 81% were either married or cohabiting, with the majority being of Han nationality (90%) and residents of rural areas (66%). On average, the participants exercise for 66 min daily. 

The elderly migrants possessed poor socioeconomic status. Sixty-two percent had primary school education or below. The average household income of the migrants was 1970 yuan, whereas the average wage was 4570 yuan in the same period. Although the share of public expenditure in gross domestic product (GDP) was above 10%, less than 50% of the respondents reported a healthy status. This could be deduced because only 10% of the migrants were fully insured. 

Comparing those who move for looking after children, we observed that the elderly migrants who move to find a job, for retirement or for keeping fit tended to be mostly male, older, earning lesser income, and less likely to consider themselves as being in good health. 

Table 2 presents the association between the individual and local socioeconomic status of migrants and their health. A positive correlation between individual income and health status was observed. A one-percent higher household income per capita was associated with a 30% lower likelihood of poor health (Table 2, Model 1). In addition, a one-percent higher average area-level wage was related to a 31% lower likelihood of having an unhealthy status. However, a one-percent higher share of the local public expenditure in GDP was associated with a three-percent higher incidence of poor health (Table 2, Model 2). Given the same household income, migrants living in the area with a one-percent higher average wage were related to a 41% higher probability of having an unhealthy status (Table 2, Model 3). 

Table 3 and Table 4 present the analyses stratified by the reasons for migration. The basic results were observed to be consistent. Household income per capita was still negatively associated with poor health outcomes, whereas the local socioeconomic status was not significantly related to the health of migrants moving to look after children. 

## 4. Discussion

This study estimates the association between the individual and local socioeconomic status of elderly migrants and their health, using a subsample of the nationally representative data for the elderly migrants in China; to my knowledge, it is the first study to do so. We observed that both individual and local socioeconomic statuses were important predictors of the health status of the elderly migrants. In particular, a higher individual and local socioeconomic status was related to a significantly lower probability of being unhealthy. However, the elderly migrants’ health status benefited little from the increasing public service supply. 

The positive association between individual income and health outcome is reasonably expected to continue among the Chinese elderly migration population because they do not receive social benefits due to various institutional barriers (e.g., the *hukou* system), and have to rely more on individual sources for medical services [23,24,25]. The basic health insurance system in China is closely related to local residents’ registration (*hukou*), and the migration population clearly had limited access to local health insurance until very recently [26,27]. In 2009, the central government issued and carried out new policies that allowed transferring the individual health insurance account accumulation when moving to another residence [27]. However, the new polices deprived migrants of the accumulation from employers’ contribution to the social pooling account during this transfer process. Considering the risk of losing the accumulation from employers’ contribution, combined with the fact that the migrants are usually highly mobile, it is unlikely for migrants to transfer their health insurance accounts to local areas [28]. The basic health insurance covers 95% of the total population, while almost nine percent of the migration people are yet to be covered by any insurance policy; and only less than 10% enjoy local insurance [29]. Medical expenses cannot be reimbursed by the local medical insurance institutions for migrants without local insurance. Consequently, access to healthcare and utilization of healthcare for the migrants continues to confront barriers. Even if the public medical service provision increases, migrants may not be entitled to enjoy these services. The discrimination of national policies on migration deprived the elderly migrants of their access to the development dividend, and may also provide a possible explanation for the results of this study, which indicated that public service provisions were not significantly related to health [30]. 

Although elderly migrants’ health benefited little from the public medical service provision, their health was positively related to local economic development. First, an increasing discretionary income, resulting from local economic development, leads to improved ability to invest in health [31]. Migrants are calculated to have contributed 16% to the GDP growth in China in the last three decades. In addition, the per capita disposable income of migrant workers increased by 10% than that in the previous year (2014) [32]. Second, a long-term perspective arises along with the predicted local economic development, resulting in expanding health investment. A preference toward the future constrains the inclination of seeking short-term rewards from unhealthy behavior such as smoking and alcohol addiction, and also values the long-term consequences of health investment as well as the health risks related to unhealthy behavior [33]. However, such a connection is based on the assumption that economic development translates into individual household income. If individual income could not benefit from a local wage increase, then this increase is associated with a worsening health outcome, which is consistent with the relative income hypothesis [34]. For migrants moving for looking after their children, their income and social benefits were only slightly related to the local socioeconomic status, and the results of this study found no significant association between the local socioeconomic status and self-reported health.

The findings revealed that individual socioeconomic status and local economic development benefiting migrants would be vital for improving their health status. Thus, multilevel approaches should be emphasized to improve the elderly migrants’ health. More specifically, policy interventions should not only improve the individual socioeconomic status but also make the local economic status livable for migrants. To achieve the latter goal, the Chinese government needs to establish comprehensive, balanced, and indiscriminatory national policies on migration. First, the Chinese government should work to eliminate discriminatory policies and reduce the socioeconomic costs of migration, such as by facilitating the transfer of remittances and lowering the costs of such transfers. On the other hand, the Chinese government should provide equal development opportunities and should share results. 

Some limitations must be considered when interpreting the results of this study, including the endogenous issue. Migrants move to places with particular local infrastructure and income levels to realize optimal health, in which case the correlations shown in the paper may lead to a simultaneity issue [35]. However, when the sample was stratified based on move reasons, the results were consistent, thus indicating the validity of these results to some extent. In addition, the indicator to measure health outcomes is a retrospective self-evaluation, which may lead to measurement error. However, self-reported health is a reliable variable and its validation has been proved as providing a good summary measure of the overall health including respiratory diseases, heart disease, diabetes, headaches and migraines, ear infections, and stomach problems [19]. 

## 5. Conclusions

In China, an increasing number of families migrate with their elderly members. This is the first study to quantify the relationship between individual and local socioeconomic status and health outcomes based on a large representative sample of Chinese elderly migrants. The findings indicate that elderly migrants’ health benefited more from individual income and local economic development, rather than from local public service provision. This study highlights the importance of multilevel and non-discriminatory policies between migrants and local residents. Health promotion strategies that create a livable local status as well as enhance individual socioeconomic status should also be considered to improve migrants’ health. 

## Figures and Tables

**Table 1:** Statistical description.

Variables	All *N* = 11,111	Moving for Job, for Retirement, for Keeping Fit, or Other Reasons *N* = 7364	Moving for Looking after Children *N* = 3737
**Health Outcomes**			
Self-reported healthy (%)	44.07	40.73	50.74
Self-reported basic healthy (%)	44.5	45.23	43.05
Self-reported unhealthy (%)	11.43	14.04	6.21
Household income ^#^ (yuan)	1969.95 (4801.99)	1851.10 (3094.12)	2205.65 (7866.21)
**Local Socioeconomic Status**			
Average wage ^#^ (yuan)	4570 (1398.26)	4370.66 (1350.73)	4968.69 (1483.48)
The share of public expenditure in GDP ^#^ (%)	10.19 (5.54)	9.72 (5.29)	11.13 (5.98)
**Migrate Characteristics**			
Intra-urban mobility (%)	42.81	39.14	50.16
**Migrate Reasons**			
Moving for job or for others reasons (%)	32.51	48.76	0
Moving with their family members mentfor aged or for keeping fit (%)	34.19	51.24	0
Looking after children (%)	33.3	0	100
**Individual Characteristics**			
Age ^#^ (year)	66.81 (6.33)	67.49 (7.17)	65.46 (4.83)
Married (%)	80.77	79.95	82.41
Rural hukou (%)	66.26	66.11	66.56
Male (%)	51.87	56/65	42.32
Han nationality (%)	90.43	90.17	90.96
Average daily exercise time ^#^ (minutes)	66.08 (45.97)	66.09 (47.20)	68.99 (44.74)
**Insurance Status**			
Uninsured (%)	14.81	15.48	13.48
Under-insured (%)	75.43	73.38	79.53
Fully insured (%)	9.76	11.15	6.99
**Education Attainment**			
Primary school and below (%)	61.59	61.38	62.01
High school (%)	24.49	24.79	23.90
College and above (%)	13.92	13.83	14.10

^#^: indicates continuous variable; Mean (Standard deviation) of continuous variables is shown.

**Table 2:** Association between the individual and local socioeconomic status of migrants and their health.

	Model 1	Model 2	Model 3
	OR (95 CIs)	OR (95 CIs)	OR (95 CIs)
Household income	0.695 ***	0.781 ***	0.015 ***
	(0.659–0.732)	(0.733–0.831)	(0.001–0.187)
Average wage		0.588 **	0.033 ***
		(0.354–0.976)	(0.004–0.253)
Share of public expenditure in GDP		1.030 **	0.951
		(1.005–1.055)	(0.860–1.053)
Household income * Average wage			1.412 ***
			(1.111–1.795)
Household income * Share of public expenditure in GDP			1.009
			(0.997–1.021)
Age	1.243 ***	1.265 ***	1.261 ***
	(1.131–1.366)	(1.145–1.399)	(1.144–1.390)
Age quadric	0.999 ***	0.999 ***	0.999 ***
	(0.998–1.000)	(0.998–0.999)	(0.998–0.999)
Male	1.069 *	1.078 *	1.078 **
	(0.997–1.146)	(0.999–1.164)	(1.000–1.161)
Han nationality	0.789 ***	0.973	0.883 *
	(0.700–0.890)	(0.826–1.145)	(0.763–1.022)
Married	0.956	0.926	0.943
	(0.867–1.055)	(0.834–1.029)	(0.851–1.044)
Intra-urban mobility	0.760 ***	0.741 ***	0.733 ***
	(0.706–0.819)	(0.670–0.819)	(0.672–0.800)
Move for retirement or for keeping fit	1.123 ***	1.106 **	1.135 **
	(1.029–1.227)	(1.000–1.223)	(1.031–1.250)
Moving for looking after children	1.896 ***	1.819 ***	1.959 ***
	(1.726–2.083)	(1.638–2.020)	(1.770–2.167)
High school	0.793 ***	0.758 ***	0.769 ***
	(0.727–0.866)	(0.686–0.836)	(0.700–0.846)
College and above	0.830 ***	0.795 ***	0.832 ***
	(0.741–0.930)	(0.698–0.905)	(0.735–0.943)
Rural hukou	1.024	1.027	1.046
	(0.941–1.115)	(0.932–1.133)	(0.953–1.147)
Under-insured	1.051	0.913	0.964
	(0.934–1.182)	(0.787–1.059)	(0.840–1.107)
Fully insured	1.101 **	1.094	1.044
	(0.998–1.213)	(0.976–1.227)	(0.936–1.165)
Average daily exercise time	0.922 ***	0.904 ***	0.915 ***
	(0.907–0.937)	(0.890–0.919)	(0.901–0.929)
Observations	11,111	11,111	11,111

GDP: gross domestic product; OR: odds ratios; CIs: confidence intervals; ***: *p* < 0.01; **: *p* < 0.05; *: *p* < 0.1.

**Table 3:** Association between individual and local socioeconomic status and health status among migrants moving to find a job or for retirements or for keeping fit or other reasons.

	Model 1	Model 2	Model 3
	Odds Ratio (95 CIs)	Odds Ratio (95 CIs)	Odds Ratio (95 CIs)
Household income	0.624 ***	0.690 ***	0.014 **
	(0.580–0.672)	(0.630–0.755)	(0.000–0.593)
Average wage		0.557 **	0.049 **
		(0.313–0.989)	(0.004–0.653)
Share of public expenditure in GDP		1.024 *	1.053
		(0.997–1.052)	(0.933–1.187)
Household income * Average wage			1.420 *
			(0.999–2.019)
Household income * Share of public expenditure in GDP			0.996
			(0.980–1.013)
Age	1.455 ***	1.557 ***	1.512 ***
	(1.307–1.619)	(1.387–1.749)	(1.351–1.692)
Age quadric	0.998 ***	0.997 ***	0.998 ***
	(0.997–0.999)	(0.997–0.998)	(0.997–0.998)
Male	1.063	1.063	1.062
	(0.977–1.158)	(0.967–1.170)	(0.968–1.164)
Han nationality	0.865 *	0.969	0.952
	(0.747–1.001)	(0.784–1.197)	(0.789–1.150)
Married	0.936	0.907	0.931
	(0.828–1.059)	(0.795–1.034)	(0.820–1.057)
Intra-urban mobility	0.762 ***	0.748 ***	0.730 ***
	(0.696–0.835)	(0.660–0.847)	(0.656–0.812)
Move for retirement or for keeping fit	1.711 ***	1.663 ***	1.742 ***
	(1.556–1.882)	(1.493–1.852)	(1.571–1.932)
High school	0.803 ***	0.759 ***	0.789 ***
	(0.722–0.894)	(0.671–0.860)	(0.701–0.888)
College and above	0.853 **	0.785 ***	0.865 *
	(0.743–0.979)	(0.666–0.926)	(0.739–1.013)
Rural hukou	0.918	0.932	0.923
	(0.824–1.023)	(0.821–1.057)	(0.819–1.040)
Under-insured	0.989	0.860 *	0.881
	(0.864–1.132)	(0.723–1.023)	(0.750–1.035)
Fully insured	1.119 *	1.111	1.080
	(0.993–1.261)	(0.964–1.281)	(0.944–1.235)
Average daily exercise time	0.921 ***	0.899 ***	0.913 ***
	(0.904–0.939)	(0.882–0.917)	(0.896–0.930)
Observations	7364	7364	7364

CIs: confidence intervals; ***: *p* < 0.01; **: *p* < 0.05; *: *p* < 0.1.

**Table 4:** Association between individual and local socioeconomic status and health status among the elderly migrants moving for looking after children.

	Model 1	Model 2	Model 3
	Odds Ratio (95 CIs)	Odds Ratio (95 CIs)	Odds Ratio (95 CIs)
Household income	0.722 ***	0.786 ***	0.036
	(0.660–0.789)	(0.706–0.874)	(0.000–3.006)
Average wage		0.608	0.070
		(0.317–1.165)	(0.002–2.716)
Share of public expenditure in GDP		1.030 *	0.895
		(0.998–1.064)	(0.741–1.080)
Household income * Average wage			1.293
			(0.851–1.966)
Household income * Share of public expenditure in GDP			1.016
			(0.994–1.038)
Age	0.728 ***	0.677 ***	0.719 ***
	(0.583–0.908)	(0.529–0.867)	(0.567–0.911)
Age quadric	1.003 ***	1.003 ***	1.003 ***
	(1.001–1.004)	(1.001–1.005)	(1.001–1.005)
Male	0.951	0.932	0.928
	(0.842–1.074)	(0.817–1.063)	(0.818–1.054)
Han nationality	0.704 ***	0.964	0.874
	(0.567–0.873)	(0.742–1.252)	(0.689–1.107)
Married	0.922	0.922	0.917
	(0.781–1.089)	(0.769–1.107)	(0.770–1.092)
Intra-urban mobility	0.755 ***	0.720 ***	0.755 ***
	(0.665–0.857)	(0.605–0.858)	(0.648–0.879)
High school	0.889	0.866 *	0.850 **
	(0.763–1.036)	(0.730–1.027)	(0.724–0.999)
College and above	0.943	0.966	0.942
	(0.771–1.154)	(0.776–1.202)	(0.764–1.161)
Rural hukou	1.025	1.081	1.070
	(0.885–1.188)	(0.917–1.275)	(0.915–1.250)
Under-insured	1.235 *	1.124	1.188
	(0.968–1.575)	(0.837–1.511)	(0.906–1.558)
Fully insured	1.074	1.033	1.014
	(0.906–1.274)	(0.848–1.259)	(0.840–1.225)
Average daily exercise time	0.941 ***	0.927 ***	0.937 ***
	(0.910–0.972)	(0.898–0.958)	(0.909–0.966)
Observations	3737	3737	3737

CIs: confidence intervals; ***: *p* < 0.01; **: *p* < 0.05; *: *p* < 0.1.
